# Increased planting density of Chinese milk vetch (*Astragalus sinicus*) weakens phosphorus uptake advantage by rapeseed (*Brassica napus*) in a mixed cropping system

**DOI:** 10.1093/aobpla/plz033

**Published:** 2019-06-22

**Authors:** Deshan Zhang, Hongbo Li, Zishi Fu, Shumei Cai, Sixin Xu, Haitao Zhu, Jianbo Shen

**Affiliations:** 1 Institute of Ecological Environment Protection Research, Shanghai Academy of Agricultural Sciences, Shanghai Key Laboratory of Protected Horticultural Technology, Shanghai Low-carbon Agricultural Technical Engineering Research Center, Shanghai, China; 2 Institute of Environment and Sustainable Development in Agriculture, Chinese Academy of Agricultural Sciences, Beijing, China; 3 Department of Plant Nutrition, China Agricultural University, Key Laboratory of Plant-Soil Interactions, Ministry of Education, Beijing, China

**Keywords:** intercropping, P-use efficiency, rhizosphere processes, root morphology, root physiology, root–root interaction

## Abstract

Neighbouring plants can affect plant growth through altering root morphological and physiological traits, but how exactly root systems respond to neighbouring plants with varied density, determining nutrient uptake and shoot growth is poorly understood. In a pot-based experiment, rapeseed was grown alone (single rapeseed), or mixed with 3, 6, or 15 Chinese milk vetch plants. As controls, monocropped Chinese milk vetch was grown at the same planting density, 3, 6, or 15 plants per pot. Root interaction between rapeseed and Chinese milk vetch facilitated phosphorus (P) uptake in rapeseed grown with 3 plants of Chinese milk vetch. As the planting density of Chinese milk vetch in mixture increased, there was a decrease in citrate concentration and acid phosphatase activity but an increase in the total root length of Chinese milk vetch per pot, resulting in decreases in rapeseed root biomass, total root length and P uptake when rapeseed was grown with 6 or 15 Chinese milk vetch plants relative to rapeseed grown with 3 plants. These results demonstrate that the enhanced nutrient utilization induced by root interaction at low planting densities was eliminated by the increased planting density of the legume species in rapeseed/Chinese milk vetch mixed cropping system, suggesting that root/rhizosphere management through optimizing legume planting density is important for improving crop productivity and nutrient-use efficiency.

## Teaser

The positive effects induced by neighbouring legumes on nutrient uptake depended on the legume planting density in a mixed cropping system. In rapeseed/Chinese milk vetch system, the Chinese milk vetch at low planting density had a small root system but high levels of citrate and acid phosphatase exudation, thus facilitating rapeseed phosphorus (P) uptake; as the Chinese milk vetch planting density increased, there was a decrease in rapeseed P uptake due to the reduction in root exudation but root-size increase in Chinese milk vetch. Optimizing legume planting density is important for improving nutrient-use efficiency in mixed cropping system with legumes.

## Introduction

Root interaction among plants plays an important role in promoting nutrient uptake, enhancing the performance of neighbouring individuals and thus plant fitness in natural communities and agroecological systems (Hinsinger *et al.* 2011; Duchene *et al.* 2017; Jesch *et al.* 2018). Interspecific facilitation occurs when one species can modify the morphological (including root length and root biomass) or physiological plasticity (such as exudation of organic and inorganic compounds) of nearby plants, ultimately benefiting these intercropped species by effectively increasing nutrient availability (Callaway 1995; Brooker *et al.* 2015; Li *et al.* 2014). For example, phosphorus-mobilizing (P-mobilizing) crops can improve P availability for themselves and neighbouring non-P-mobilizing species through carboxylates and phosphatases, as in the case of maize intercropped with faba bean (Wang *et al.* 2007; Dissanayaka *et al.* 2015; Li *et al.* 2018).

Plants can discriminate between roots belonging to themselves and a physiologically independent individual of either the same or a different genotype, allowing them to both discriminate between sibling and non-sibling neighbours, and between neighbours belonging to different species (Semchenko *et al.* 2014; McNickle *et al.* 2015; Abakumova *et al.* 2016; Li *et al.* 2019). When neighbours are present, plants are capable of changing root response in ways that can lead to increased root proliferation (Semchenko *et al.* 2007; Mommer *et al.* 2010), decreased root growth (Schenk *et al.* 1999; Dudley and File 2007; Murphy and Dudley 2007) or no response (Litav and Harper 1967; Novoplansky 2009) as appropriate. For instance, the root growth of *Abutilon theophrasti* was significantly inhibited in the presence of the same neighbouring species (Cahill *et al.* 2010). As compared with two maize plants grown together, greater citrate concentration and acid phosphatase activity in rhizosphere soil induced by neighbouring faba bean can stimulate root growth in maize (Zhang *et al.* 2016). Neighbouring plant identity thus drives root plasticity and consequently governs nutrient uptake by plants (Cahill *et al.* 2010; Mommer *et al.* 2012; Zhang *et al.* 2016).

Mixed cropping or intercropping is widely adopted in Chinese intensive farming systems and is a good example of cropping systems that deliver yield advantages and enhance nutrient productivity due to beneficial interactions between neighbouring plants (Li *et al.* 2007; Zhang *et al.* 2010; Shen *et al.* 2011, 2013; Isbell *et al.* 2017). However, the success of this agroecological practice depends to a great extent on the environmental context, such as the crop planting density in mixed cropping systems (Hauggaard-Nielsen and Jensen 2005). For instance, Willey and Osiru (1972) demonstrated that a higher density of component crops when intercropping resulted in greater intercrop advantages in a maize/bean intercropping system. However, the dry matter production of soybean decreased as its planting density increased in a maize/soybean intercropping system (Ren *et al.* 2016). While some of the positive or negative effects of planting density on crop growth in intercropping are well documented (Rodrigo 1997; Echarte *et al.* 2011), the mechanisms underlying these complex root morphological and physiological plasticity in responding to varied planting density, and their effects on nutrient uptake, are poorly understood.

Growing mixtures of rapeseed (*Brassica napus* L.) and Chinese milk vetch (*Astragalus sinicus* L.) can improve soil fertility, increase oil production and thereby benefit farmers (Lin *et al.* 2011). Given these intercropping advantages, the rapeseed/Chinese milk vetch mixed cropping system has been popularly adopted in the paddy soil after rice harvested and is mainly distributed in provinces of Fujian, Zhejiang, Jiangxi, Jiangsu, Hu’nan, Hubei and Anhui in China (Lin *et al.* 2011; Song 2013). Rapeseed is one of the most important crops used for oil production globally, and it exhibits strong root morphological plasticity, increases exudation of carboxylates (particularly citrate) and enhances activity of phosphatase enzymes when suffering from P deficiency (Lyu *et al.* 2016; Wen *et al.* 2019). Chinese milk vetch is a leguminous crop with a small and shallow root system; and Chinese milk vetch tolerated P stress through secreting great carboxylate and acid phosphatase enzyme (Lan *et al.* 2011, 2012). Though rapeseed and Chinese milk vetch may have much in common in P capturing strategies, there is currently a lack of a general framework that can explain whether root interaction between rapeseed and Chinese milk vetch drives crop P uptake advantage in a mixture.

Relative planting density between rapeseed and Chinese milk vetch is a critical factor determining rapeseed production in practice (Zhou *et al.* 2005; Song 2013). A previous study has demonstrated that rapeseed/Chinese milk vetch improved rapeseed grain yield only when the relative seeding ratio of rapeseed to Chinese milk vetch was suitable (Song 2013; Song *et al.* 2014). Hence, an understanding of root interaction for soil P acquisition in rapeseed and Chinese milk vetch at varied planting densities, and the mechanisms regulating crop growth, is needed to maximize the intercropping advantages via optimizing the relative planting density between rapeseed and Chinese milk vetch, thereby enhancing crop yields and related economic benefits. To understand the effect on P uptake by rapeseed and Chinese milk vetch in mixture, a pot experiment was conducted to test: (i) how root interaction between rapeseed and Chinese milk vetch affects root morphological (e.g. root biomass and total root length) and physiological (e.g. citrate concentration and acid phosphatase activity) traits and consequently plant P uptake; and (ii) whether the P-acquisition mechanism in rapeseed and Chinese milk vetch depends on the planting density of Chinese milk vetch in mixture.

## Materials and Methods

### Experimental set-up

**Fig. 1.**
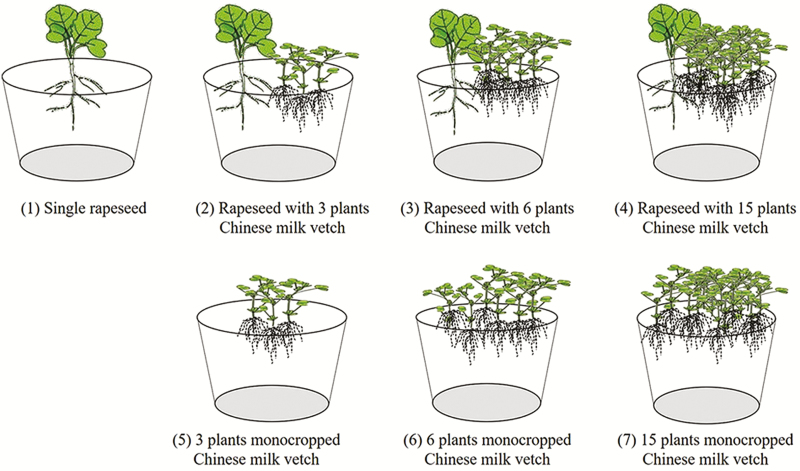
Design of the pot-experimental treatments.

To investigate how rapeseed and Chinese milk vetch root (including morphology and physiology) responds to the varied Chinese milk vetch planting density, and test the effects on P uptake, a pot-based experiment was conducted in a glasshouse with three different cropping treatments and three different Chinese milk vetch planting densities. Rapeseed was grown alone as a single species (single rapeseed treatment), or mixed with 3, 6 or 15 plants of Chinese milk vetch; corresponding to the density of Chinese milk vetch in mixture, monocropped Chinese milk vetch was at the same density with 3, 6 and 15 plants per pot (Fig. 1). There were seven total treatment combinations, with four replicates per treatment.

Soil was collected from a paddy field in Zhuanghang experimental station in Shanghai, China, air-dried and passed through a 2-mm sieve. The soil contained (per kg) 17.8 g organic carbon, 1.23 g total N, 66.55 mg available N (NO_3_^−^+NH_4_^+^), 13.8 mg NaHCO_3_-extractable P, 241 mg NH_4_Ac-K, and had pH of 6.75 (the ratio of soil to 0.01 M CaCl_2_ solution was 1:2.5). Phosphorus as Ca(H_2_PO_4_)_2_·H_2_O was applied at 20 mg kg^−1^ soil based on the P fertilizer application rate in field condition (Song 2013), corresponding with soil Olsen-*P* values of 18.9 mg kg^−1^. Pots (17 cm surface diameter, 9 cm bottom diameter and 9 cm height) were filled with 2 kg of air-dried soil. To ensure that the nutrient supply was adequate for plant growth, soil was also fertilized with basal nutrients at the following rates (mg pot^−1^): Ca(NO_3_)_2_·4H_2_O, 1687; K_2_SO_4_, 200; MgSO_4_·7H_2_O, 65; Fe-EDTA, 8.78; MnSO_4_·H_2_O, 10; ZnSO_4_·7H_2_O, 15; CuSO_4_·5H_2_O, 3; H_3_BO_3_, 2 and Na_2_MoO_4_·5H_2_O, 0.25.

The genotype of rapeseed in this study was *Brassica napus* L. Huyou19, and the genotype of Chinese milk vetch was *Astragalus sinicus* L. Yijiang. Rapeseed and Chinese milk vetch seeds were surface sterilized in 30 % v/v H_2_O_2_ for 20 min, washed with deionized water, soaked in a CaSO_4_-saturated solution for 12 h and then germinated in Petri dishes covered with wet filter paper for 1 day at 25 °C. All the pots were arranged in a completely randomized design and were re-randomized weekly during the experiment. Soil moisture was kept at 18 % (w/w) as determined gravimetrically by weighing each pot every day during the experiment.

Plants were harvested 40 days after sowing and separated into shoots and roots. Following root excavation, the soil adhering to roots was defined as rhizosphere soil and was sub-sampled for carboxylate and acid phosphatase measurements. Roots were transferred to a tube containing 50 mL of 0.2 mΜ CaCl_2_ and gently shaken to dislodge the rhizosphere soil, followed by shaking for 5–10 s to create a homogeneous suspension (Pearse *et al.* 2007). A suspension volume of 10 mL was taken by pipette to a 10-mL centrifuge tube for carboxylate analysis by high-performance liquid chromatography (HPLC), and a 0.5 μL aliquot of the suspension was placed in a 2-mL centrifuge tube for acid phosphatase measurement.

The experiment was conducted in a glasshouse at Zhuanghang Experimental Station of Shanghai Academy of Agricultural Sciences, Shanghai (30°53′N, 121°22′E). The temperature in the glasshouse was maintained at 14–19 °C during the day and 6–9 °C at night, with 10.2–11 h of daylight throughout the growth period.

### Measurements

#### Plant biomass and P uptake

Shoots were oven-dried at 105 °C for 30 min and then at 65 °C for 3 days before weighing for dry biomass determination. Phosphorus concentration in shoots was determined after digestion with a mixture of 5 mL of concentrated sulphuric acid and 8 mL of 30 % v/v H_2_O_2_. Shoot P was analysed by the molybdovanadophosphate method at 440 nm via spectrophotometry (Varian Vista–Pro CCD) as previously described (Johnson and Ulrich 1959).

According to Hedges *et al.* (1999), we used a meta-analysis method to study the correlation of effect size of oil grain yield in rapeseed with the relative planting density of Chinese milk vetch to rapeseed and to investigate the effects of Chinese milk vetch planting density on rapeseed growth (including eight papers published in the literatures, see references in Supporting Information**—Table S1**).

Roots were washed in deionized water and scanned with an EPSON root scanner at 400 dots-per-inch resolution (Epson Expression 1600 pro, Model EU-35, Japan). The total root length was analysed with the Win-RHIZO software (Regent Instruments Inc., Quebec, QC, Canada). The total root length was divided by the shoot biomass, measured as the proportion of root length-to-shoot biomass. The measurement was used to estimate how roots respond to soil environment cues (such as root interaction between rapeseed and Chinese milk vetch, and the Chinese milk vetch planting density) in order to support the shoot growth.

Carboxylates in the rhizosphere soil were analysed using a reversed-phase HPLC system according to a previous report modified from Shen *et al.* (2003) and Wang *et al.* (2010). The chromatographic separation was conducted on a 250 × 4.6 mm reversed-phase column (Alltima C18, 5 Micrometers; Alltech Associates, Inc., Deerfield, IL, USA). The mobile phase was 25 mmol L^−1^ KH_2_PO_4_ (pH 2.25) with a flow rate of 1 mL min^−1^ at 31 °C. Detection of carboxylates was carried out at 214 nm.

To determine the activity of acid phosphatase in the rhizosphere soil, 0.5 mL aliquots of soil suspensions were transferred into a 2-mL Eppendorf vial with 0.4 mL sodium acetate buffer and 0.1 mL *p*-nitrophenyl phosphate substrate added. Vials were incubated at 30 °C for 60 min, and the reaction was terminated by adding 0.5 mL of 0.5 M NaOH. Absorption was measured at 405 nm (Alvey *et al.* 2000).

### Statistical analysis

We used the one-way analysis of variance to test the effects of Chinese milk vetch planting density on shoot biomass, P uptake, root morphological (including root biomass and root length) and physiological (including citrate concentration and acid phosphatase activity in rhizosphere soil) traits; when appropriate, post hoc means comparisons were conducted using the Tukey’s test at a 5 % probability level (*P* ≤ 0.05) with the SPSS statistical software (SPSS version 23.0, IBM SPSS Inc., Chicago, IL, USA). Student’s *t*-tests were conducted to detect significant differences in rapeseed or Chinese milk vetch between the mixed cropping systems and the corresponding single (for rapeseed) or monocropping (for Chinese milk vetch) controls.

## Results

**Fig. 2.**
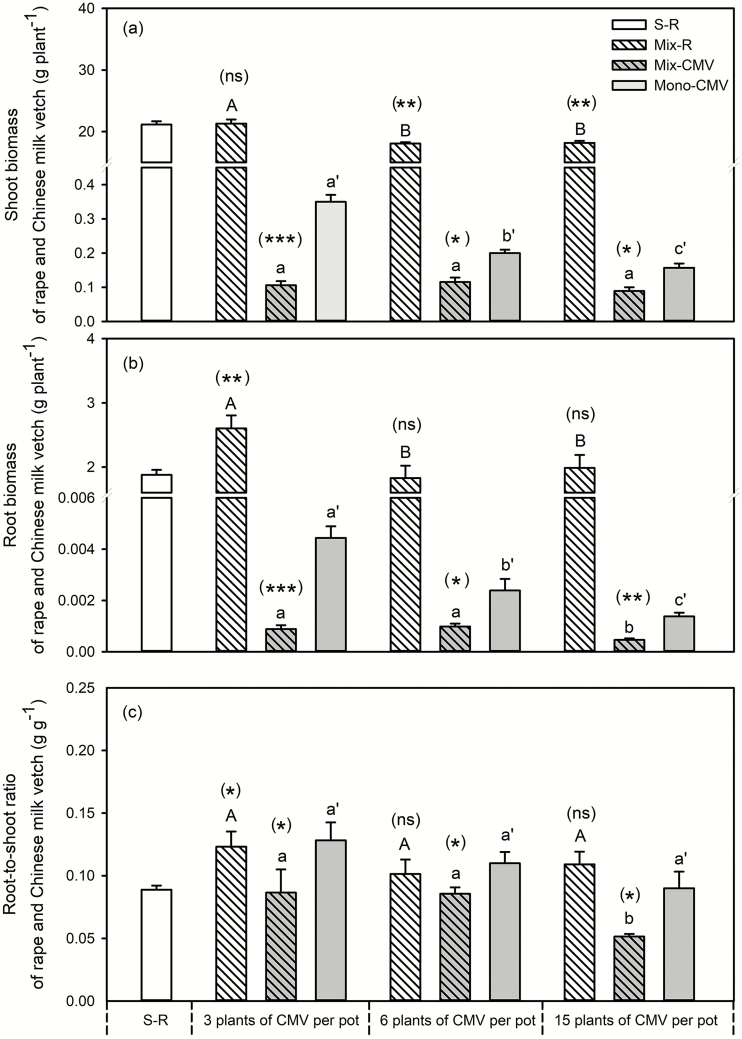
Effects of Chinese milk vetch planting density on plant shoot biomass (a), root biomass (b) and root-to-shoot ratio (c) in rapeseed/Chinese milk vetch mixture. S-R: single rapeseed; Mix-R: rapeseed in mixture; Mix-CMV: Chinese milk vetch in mixture; Mono-CMV: Chinese milk vetch in a monocropping system. Each value is the mean of four replicates (+SE). Different capital letters denote significant differences among rapeseed plants mixed with 3, 6 or 15 Chinese milk vetch plants per pot (*P* ≤ 0.05), and different lowercase letters denote significant difference in Chinese milk vetch. Different lowercase with single quotes (‘) denotes significant differences in monocropped Chinese milk vetch plants. Significant differences in shoot biomass of rapeseed or Chinese milk vetch between the single (for rapeseed) or monocropping (for Chinese milk vetch) and mixed systems were assessed by Student’s *t*-test, with the brackets above each column indicating the rapeseed and Chinese milk vetch quantities in a given mixture, *P ≤ 0.05, **P < 0.01, ***P < 0.001, ns = not significant.

The effects of root interactions between rapeseed and Chinese milk vetch on shoot biomass were dependent on the planting density of Chinese milk vetch in mixture (Fig. 2a). The shoot biomass for rapeseed grown with 3 plants of Chinese milk vetch was similar to single rapeseed; in rapeseed grown with 6 and 15 Chinese milk vetch plants, the shoot biomass was lower than single plant. Compared with Chinese milk vetch in monocropping system, the shoot biomass of Chinese milk vetch was lower in mixture. The increased planting density in Chinese milk vetch had little effect on rapeseed shoot growth in mixture, being greater in rapeseed with 3 plants Chinese milk vetch than that with 6 or 15 plants, whereas there was no difference between rapeseed with 6 and 15 plants. Although the shoot biomass of Chinese milk vetch in monocropped samples decreased with the increase in Chinese milk vetch planting density, it remained similar in mixed cropping samples, regardless of the planting density.

Compared with single rapeseed, root biomass in rapeseed grown with 3 plants Chinese milk vetch was greater, while it was similar for rapeseed grown with 6 and 15 plants (Fig. 2b). In rapeseed/Chinese milk vetch mixture, the root biomass of Chinese milk vetch was significantly lower than that in monocropping treatments. Root biomass in rapeseed and Chinese milk vetch in responding to Chinese milk vetch planting density showed similar trends to those observed for shoot biomass, except that root biomass of Chinese milk vetch varied with Chinese milk vetch planting density in mixed samples. In rapeseed growing with 15 plants of Chinese milk vetch mixture, the root biomass of Chinese milk vetch was lower than that containing 3 and 6 plants per pot; however, there was no difference between the root biomass in the samples with 3 and 6 plants of Chinese milk vetch.

The plant root-to-shoot ratio was significantly affected by root interaction between rapeseed and Chinese milk vetch and also by the planting density of Chinese milk vetch (Fig. 2c). Compared with single plant, the root-to-shoot ratio for rapeseed grown with 3 Chinese milk vetch plants was greater, while there was no difference for rapeseed grown with 6 and 15 Chinese milk vetch plants from single treatment. The presence of rapeseed decreased the root-to-shoot ratio in Chinese milk vetch compared with that in monocropping treatments, regardless of Chinese milk vetch planting density. The planting density of Chinese milk vetch had little effect on the root-to-shoot ratio of rapeseed in mixture, or on the monocropped Chinese milk vetch. For Chinese milk vetch in mixture, the root-to-shoot ratio was significantly influenced by Chinese milk vetch planting density, being similar at densities containing 3 or 6 plants Chinese milk vetch per pot, followed by the treatment containing 15 plants.

**Fig. 3.**
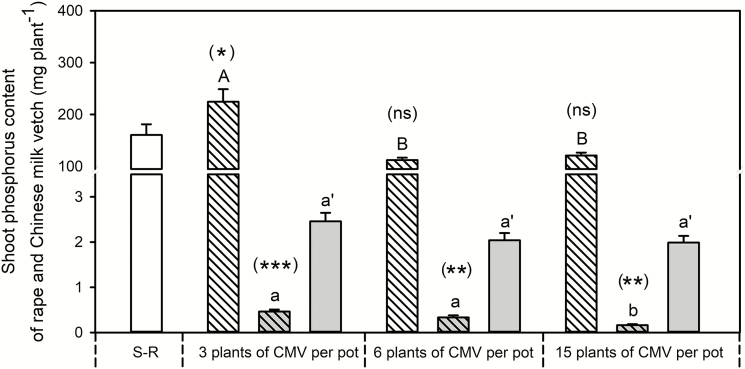
Effects of Chinese milk vetch planting density on plant shoot P content in rapeseed/Chinese milk vetch mixture. S-R: single rapeseed; Mix-R: rapeseed in mixture; Mix-CMV: Chinese milk vetch in mixture; Mono-CMV: Chinese milk vetch in a monocropping system. Each value is the mean of four replicates (+SE). Different capital letters denote significant differences among rapeseed plants mixed with 3, 6 or 15 Chinese milk vetch plants per pot (*P* ≤ 0.05), and different lowercase letters denote significant difference in Chinese milk vetch. Different lowercase with single quotes (‘) denotes significant differences in monocropped Chinese milk vetch plants. Significant differences in shoot biomass of rapeseed or Chinese milk vetch between the single (for rapeseed) or monocropping (for Chinese milk vetch) and mixed systems were assessed by Student’s *t*-test, with the brackets above each column indicating the rapeseed and Chinese milk vetch quantities in a given mixture, *P ≤ 0.05, **P < 0.01, ***P < 0.001, ns = not significant.

The root interaction between rapeseed and Chinese milk vetch, and the Chinese milk vetch planting density had significant effects on shoot P content in rapeseed and Chinese milk vetch (Fig. 3). Shoot P content grown with 3 Chinese milk vetch plants was greater than single rapeseed; while rapeseed grown with 6 and 15 Chinese milk vetch plants showed similar shoot P content to single rapeseed. For Chinese milk vetch, the shoot P content was significantly lower in mixture than in monocropping samples. With the increase of Chinese milk vetch planting density in mixture, shoot P content in rapeseed decreased, resulting in that rapeseed P uptake grown with 3 Chinese milk vetch plants was greater than that grown with 6 and 15 plants, whereas there was no difference in treatments between rapeseed grown with 6 and 15 Chinese milk vetch plants. The shoot P content for Chinese milk vetch in mixture decreased with an increase in Chinese milk vetch plants; however, the planting density had no impact on shoot P content in monocropped Chinese milk vetch.

The correlation of oil grain yield in rapeseed with the relative planting density of Chinese milk vetch to rapeseed based on field experiments reported in the literature was calculated to examine the effects of Chinese milk vetch planting density on rapeseed productivity **[see Supporting Information—****Fig. S1****]**. The mixed cropping system between rapeseed and Chinese milk vetch could improve oil grain yield of rapeseed when the planting ratio was reasonable. However, with the increase of relative planting density of Chinese milk vetch to rapeseed, the oil grain yield in rapeseed decreased significantly.

**Fig. 4.**
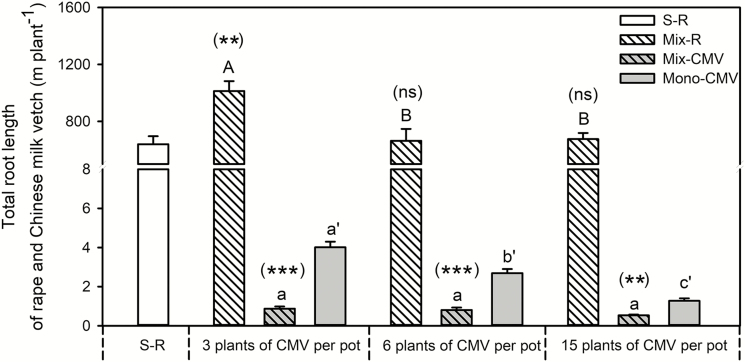
Effects of Chinese milk vetch planting density on plant total root length in rapeseed/Chinese milk vetch mixture. S-R: single rapeseed; Mix-R: rapeseed in mixture; Mix-CMV: Chinese milk vetch in mixture; Mono-CMV: Chinese milk vetch in a monocropping system. Each value is the mean of four replicates (+SE). Different capital letters denote significant differences among rapeseed plants mixed with 3, 6 or 15 Chinese milk vetch plants per pot (*P* ≤ 0.05), and different lowercase letters denote significant difference in Chinese milk vetch. Different lowercase with single quotes (‘) denotes significant differences in monocropped Chinese milk vetch plants. Significant differences in shoot biomass of rapeseed or Chinese milk vetch between the single (for rapeseed) or monocropping (for Chinese milk vetch) and mixed systems were assessed by Student’s *t*-test, with the brackets above each column indicating the rapeseed and Chinese milk vetch quantities in a given mixture, *P ≤ 0.05, **P < 0.01, ***P < 0.001, ns = not significant.

Total root length (Fig. 4) and the proportion of total root length-to-shoot biomass **[see Supporting Information—****Fig. S1****]** were calculated to examine how root morphology responds to root interaction between rapeseed and Chinese milk vetch, and the varied planting density of Chinese milk vetch. Compared with single rapeseed, total rapeseed root length (Fig. 4) was greater in the treatment containing 3 Chinese milk vetch plants, whereas it did not vary for rapeseed grown with 6 or 15 plants of Chinese milk vetch. The root length per Chinese milk vetch in the mixed cropping samples was lower than in the monocropping controls, regardless of the planting density. Overall, total rapeseed root length decreased with an increase in the density of Chinese milk vetch. Rapeseed root length was greater when grown with 3 Chinese milk vetch plants relative to that grown with 6 or 15 plants, although there was no significant difference between the 6 and 15 plants samples. In addition, root length per Chinese milk vetch in mixed cropping system was not affected by the planting density, however, it decreased significantly with increasing planting density of Chinese milk vetch in the monocropping samples.

The proportion of total root length-to-shoot biomass was calculated to estimate how root respond to soil environment cues (such as root interaction between rapeseed and Chinese milk vetch, and the Chinese milk vetch planting density) to support the shoot growth **[see****Supporting Information—Fig. S1****]**. The rapeseed in the mixed cropping system showed a greater proportion of root length per shoot biomass in samples grown with 3 Chinese milk vetch plants than in samples containing a single rapeseed plant or rapeseed grown with 6 or 15 Chinese milk vetch plants. The presence of rapeseed and the planting density of Chinese milk vetch had no effect on the proportion of total root length-to-shoot biomass in Chinese milk vetch in a mixed cropping system.

**Fig. 5.**
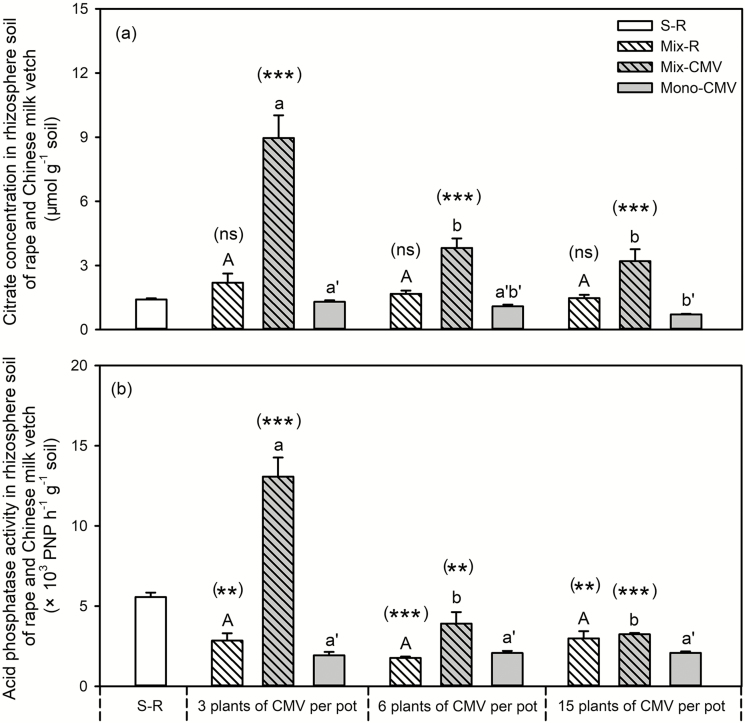
Effects of Chinese milk vetch planting density on citrate concentrations (a) and acid phosphatase activity (b) in the rhizosphere of rapeseed and Chinese milk vetch in mixture. S-R: single rapeseed; Mix-R: rapeseed in mixture; Mix-CMV: Chinese milk vetch in mixture; Mono-CMV: Chinese milk vetch in a monocropping system. Each value is the mean of four replicates (+SE). Different capital letters denote significant differences among rapeseed plants mixed with 3, 6 or 15 Chinese milk vetch plants per pot (*P* ≤ 0.05), and different lowercase letters denote significant difference in Chinese milk vetch. Different lowercase with single quotes (‘) denotes significant differences in monocropped Chinese milk vetch plants. Significant differences in shoot biomass of rapeseed or Chinese milk vetch between the single (for rapeseed) or monocropping (for Chinese milk vetch) and mixed systems were assessed by Student’s *t*-test, with the brackets above each column indicating the rapeseed and Chinese milk vetch quantities in a given mixture, **P < 0.01, ***P < 0.001, ns = not significant.

Citrate concentrations in rhizosphere soil around rapeseed were not affected by the presence or the planting density of Chinese milk vetch (Fig. 5a). The acid phosphatase activity in rapeseed rhizosphere soil from mixed cropping samples was lower than that of the single rapeseed, but there were no differences among the different mixed cropping samples with regard to Chinese milk vetch planting density (Fig. 5b). For root exudates in the rhizosphere soil of the Chinese milk vetch in monocropping system, the planting density of Chinese milk vetch had limited effect on citrate concentrations and acid phosphatase activity in the rhizosphere soil (Fig. 5), with a slight decrease in citrate concentrations in the samples with 15 Chinese milk vetch plants per pot than with 3 plants (Fig. 5a). Compared with monocropped Chinese milk vetch, citrate secretion and acid phosphatase activity in the rhizosphere soil around Chinese milk vetch in the mixed cropping samples were improved significantly, particularly in the samples with 3 plants per pot, with some improvement in the samples containing 6 and 15 Chinese milk vetch plants per pot (Fig. 5).

## Discussion

In the present study, rapeseed and Chinese milk vetch with different densities in a mixed cropping scenario were compared with rapeseed alone or Chinese milk vetch monocropped controls to assess P utilization driven by root interactions between rapeseed and Chinese milk vetch. In contrast to previous results suggesting that any neighbouring plants would potentially represent competition (Gersani *et al.* 1998; Cahill *et al.* 2010; Mommer *et al.* 2012), our findings indicated that neighbouring Chinese milk vetch planting at a low density improved P uptake by rapeseed when compared with single rapeseed, although there was decrease in Chinese milk vetch P uptake (Fig. 3). Shoot P uptake in rapeseed is improved when plants are grown with 3 Chinese milk vetch plants; however, further increase in the number of Chinese milk vetch plants decreases shoot P nutrition and weakens the mixed cropping benefits for rapeseed (Fig. 3). The results are consistent with previous studies; for example, increasing okra planting density has been found to reduce the yield of intercropped maize (Muoneke and Asiegbu 1997; Mushayabasa *et al.* 2014), and increasing the density of maize or sunflower crops has been shown to lead to a corresponding decrease in the land equivalent ratio index for these two crops (Echarte *et al.* 2011). In addition, the meta-analysis results suggested that rapeseed grain yield was negatively related with the planting density of Chinese milk vetch in mixed cropping system **[see** Supporting Information**—****Fig. S1****]**. The results showed in the present study thus suggest that Chinese milk vetch can be beneficial to rapeseed P uptake, or can have no effect, being closely related to the planting ratio between rapeseed and Chinese milk vetch in a mixture.

Nutrient uptake and shoot growth were consistent with root biomass allocation (Cahill *et al.* 2010; Mommer *et al.* 2012) and root length (Zhang *et al.* 2016). The greater root system size is associated with a higher competitive ability (Ravenek *et al.* 2016; Semchenko *et al.* 2018). In the present experiment, the significantly higher root biomass and root length in rapeseed than Chinese milk vetch may trigger the great competitive ability for acquiring P from soil. Furthermore, the varied planting density of Chinese milk vetch altered root morphology and physiology, and thus P uptake by rapeseed and Chinese milk vetch in mixture. Plants can sense and coordinate their root growth based on available soil volume (Hess and de Kroon 2007; Maina *et al.* 2002). Although root length per Chinese milk vetch in mixed cropping samples was similar, the total root length of Chinese milk vetch per pot was four times greater in treatment with 15 plants Chinese milk vetch than that of samples with 3 plants. As dense planting restricts lateral root extension (Gersani *et al.* 1998; Shao *et al.* 2018), the smaller root system of Chinese milk vetch planted at 3 plants per pot, therefore provided more space for rapeseed roots, leading to greater root length in rapeseed grown with 3 plants Chinese milk vetch than with 6 or 15 plants (Fig.4; see Supporting Information—Fig. S2).

In addition, compared with Chinese milk vetch in monocropping controls, the presence of rapeseed improved citrate concentration and acid phosphatase activity in rhizosphere soil around Chinese milk vetch root in mixture, particularly when planting at 3 plants per pot (Fig. 5). The high physiological plasticity of Chinese milk vetch in terms of the exudation of carboxylates (Fig. 5a) and acid phosphatase (Fig. 5b) could mobilize soil P to provide a P supply for the neighbouring rapeseed (Hinsinger *et al.* 2011; Brooker *et al*. 2015; Li *et al.* 2014). Roots could exhibit morphological plasticity in response to the physiological traits induced by neighbouring species (Zhang *et al.* 2016); and rapeseed is a species that primarily relies upon strong morphological plasticity to maintain biomass production under variable P supply (e.g. root length) (Figs 4 and 5; see Supporting Information—Fig. S2; see also Marschner *et al.* 2007; Pearse *et al.* 2007; Zhang *et al.* 2009; Lyu *et al.* 2016). Compared with the mixture with 6 and 15 plants Chinese milk vetch, the greater root exudates (Fig. 5) by Chinese milk vetch growing at 3 plants per pot stimulated rapeseed root proliferation (Fig. 4; see **Supporting Information—Fig. S2**), and consequently enhanced P uptake (Fig. 3). Briefly, the increased planting density of Chinese milk vetch reduced the soil volume for rapeseed root, decreased carboxylate exudation and acid phosphatase activity in rhizosphere soil, and thereby inhibited rapeseed root proliferation in order to take up P from soil. The results demonstrated that root interactions between rapeseed and Chinese milk vetch could facilitate rapeseed P uptake; however, the positive effect would be reversed if the planting density of Chinese milk vetch was too great.

In situations in which cohabiting productive species are highly complementary in nutrient-use efficiency, the determination of the optimal plant population density necessary to maximize productivity is a major agronomic route to achieving optimal yields in a mixed cropping system (Muoneke and Asiegbu 1997; Echarte *et al.* 2011; Ren *et al.* 2016). A primary challenge for researchers is in understanding the processes and mechanisms underpinning mixed cropping systems and consequent productivity. Studying the root–rhizosphere interactions in the rapeseed/Chinese milk vetch is thus important for developing strategies for rhizosphere management through optimizing relative planting density of crops in mixed or intercropping systems in order to increase crop productivity and nutrient-use efficiency. Although these results suggested that a suitable relative planting density would improve crop P uptake and oil grain yield (Fig. 3; see Supporting Information—Fig. S1), other information should be considered in predicting the crop growth advantages driven by root–rhizosphere interactions, since the yield of each component depends on light competition above ground, nitrogen application, water irrigation and other related factors (Willey and Osiru 1972; Zhang *et al.* 2010; Mommer *et al.* 2016). For example, rhizobia can assimilate nitrogen through biological nitrogen fixation, but also solubilize phosphorus (Alikhani *et al.* 2006; Sridevi and Mallaiah 2009). As nitrogen fertilizer application in the present experiment, the biological nitrogen fixation may have little effect on P uptake advantage in rapeseed/Chinese milk vetch mixed cropping system. However, future research should also thus consider the mechanisms underlying how to enhance crop productivity through improving nitrogen management in a mixed cropping system with legume species. Such knowledge has the potential to allow for the manipulation of mixed cropping systems to maximize desired outcomes such as food production, landscape quality or biodiversity conservation, which could promote wider adoption of diversified systems.

## Conclusions

In a rapeseed and Chinese milk vetch mixed cropping system, Chinese milk vetch plants modified root biomass and total root length in rapeseed, further influencing rapeseed P uptake. However, the effect of root interactions between rapeseed and Chinese milk vetch on P uptake was correlated with the planting density of Chinese milk vetch in the mixed cropping samples. Rapeseed exhibited relatively strong morphological enhancements (total root length and root biomass) in response to Chinese milk vetch at low planting density (3 plants Chinese milk vetch in mixture per pot), due to the small root system but great root exudates in relation to P mobilization. As the planting density of Chinese milk vetch further increased, the total root length per pot increased, but root exudates in the rhizosphere soil around Chinese milk vetch decreased. Compared with Chinese milk vetch at low planting density, the variation in terms of root-size increase and root exudates decrease of Chinese milk vetch at high planting density inhibited root proliferation in rapeseed. Hence, rapeseed P uptake and consequently the shoot biomass decreased significantly with the increase planting density of Chinese milk vetch in rapeseed/Chinese milk vetch mixed cropping system. Root–rhizosphere interactions induced by legume species can facilitate nutrient uptake by crops in a mixed cropping system; however, the nutrient utilization advantages would be eliminated with increased legume planting density. The study thus offers new insights into root–rhizosphere interactions in a mixed cropping system with legume species planted at different densities, which is important for the development of strategies for rhizosphere management as a means of optimizing the planting density of the legumes to increase crop productivity and nutrient-use efficiency.

## Supporting Information

The following additional information is available in the online version of this article —

Fig. S1 The correlation of oil grain yield in rapeseed with the relative planting density of Chinese milk vetch to rapeseed based on field experiments reported in the literature.

Fig. S2 Effects of planting density (3 plants, 6 plants and 15 plants per pot) of Chinese milk vetch on proportion of total root length-to-shoot biomass in rapeseed and Chinese milk vetch in mixture. 

Table S1 The effects of relative planting density of Chinese milk vetch to rapeseed on effect size of oil grain yield in rapeseed based on field experiments reported in the literature.

plz033_suppl_Supplementary_Material

plz033_suppl_Supplementary_Data

## Sources of Funding

This study was supported by the National Natural Science Foundation of China (31801946), National Key Research and Development Program of China (2017YFD0200200), Ningxia Key R&D Program (2019BBF02026), and Shanghai Science and Technology Development Fund (18295810500).

## Conflict of Interest

None declared.

## Contributions by the Authors

D.Z. and H.Z. designed research; D.Z., H.L. and H.Z. performed research; D.Z., Z.F., S.X. and S.C. collected and analysed data; D.Z., H.Z., Z.F., S.X. S.C. and J.S. wrote the paper. All authors read and approved the final manuscript.
