# Supplementary material for: Increased planting density of Chinese milk vetch (Astragalus sinicus) weakens phosphorus uptake advantage by rapeseed (Brassica napus) in a mixed cropping system
Source: AoB Plants. 2019 Jun 22;11(4):plz033. doi: 10.1093/aobpla/plz033 (PMC6605628; doi:10.1093/aobpla/plz033)
Supplement: plz033_suppl_Supplementary_Material [file plz033_suppl_supplementary_material.docx]

**Supplementary material for**

**Title:** Increased planting density of Chinese milk vetch (*Astragalus sinicus* L.) weakens phosphorus uptake advantage by rapeseed (*Brassica napus* L.) in mixed cropping system

Authors：Deshan Zhang, Hongbo Li, Zishi Fu, Shumei Cai, Sixin Xu, Haitao Zhu and Jianbo Shen

The following Supporting Information is available for this article:

Fig. S1 The correlation of oil grain yield in rapeseed with the relative planting density of Chinese milk vetch to rapeseed based on field experiments reported in the literature.

Fig. S2 Effects of planting density (3 plants, 6 plants and 15 plants per pot) of Chinese milk vetch on proportion of total root length-to-shoot biomass in rapeseed and Chinese milk vetch in mixture.

Table S1 The effects of relative planting density of Chinese milk vetch to rapeseed on effect size of oil grain yield in rapeseed based on field experiments reported in the literature.


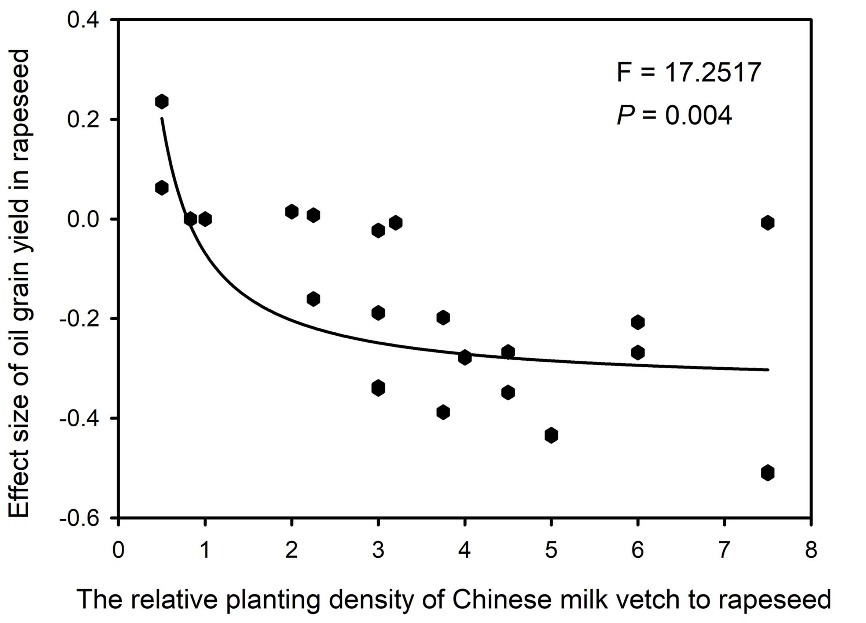


Fig. S1 The correlation of the effect size of oil grain yield in rapeseed with the relative planting density of Chinese milk vetch to rapeseed based on field experiments reported in the literature (1-8 in reference). For all studies, the data, including the mean, standard deviation, and replicate number, pertaining to oil grain yield in the control (rapeseed monocropping) and treatment (rapeseed/Chinese milk vetch mixed cropping) conditions were collected. The natural log-transformed response ratio [ln(RR)], defined as the “effect size”, which is the mean of the treatment (in rapeseed/Chinese milk vetch mixed cropping system) divided by the mean of the control (in rapeseed monocropping treatments) (Hedges *et al.* 1999). The Y-axis reflects the effect size of oil grain yield in rapeseed/Chinese milk vetch mixed cropping systems; the X-axis reflects the relative planting density of Chinese milk vetch to rapeseed in field experiments reported in the literatures.


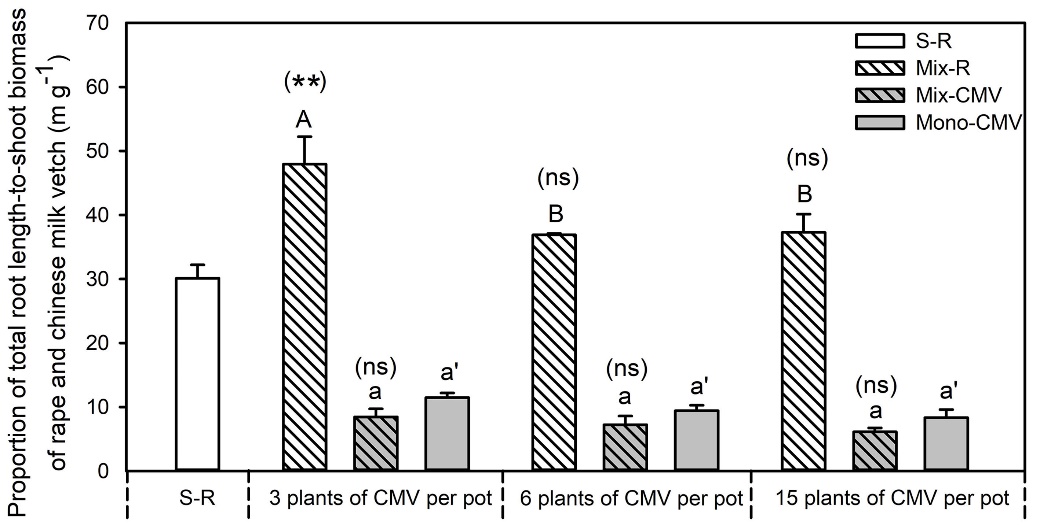


Fig. S2 Effects of planting density (3 plants, 6 plants and 15 plants per pot) of Chinese milk vetch on proportion of total root length-to-shoot biomass in rapeseed and Chinese milk vetch in mixture. S-R: single rapeseed; Mix-R: rapeseed in mixture; Mix-CMV: Chinese milk vetch in mixture; Mono-CMV: Chinese milk vetch in a monocropping system. Each value is the mean of four replicates (+SE). Different capital letters denote significant differences among rapeseed plants mixed with 3, 6, or 15 Chinese milk vetch plants per pot (*P* ≤0.05), and different lowercase letters denote significant difference in Chinese milk vetch. Different lowercase with single quotes ( ’ ) denote significant differences in monocropped Chinese milk vetch plants. Significant differences in shoot biomass of rapeseed or Chinese milk vetch between the single (for rapeseed) or monocropping (for Chinese milk vetch) and mixed systems were assessed by Student’s t-test, with the brackets above each column indicating the rapeseed and Chinese milk vetch quantities in a given mixture, ** *P* <0.01, ns= not significant.

Table S1 The effects of relative planting density of Chinese milk vetch to rapeseed on effect size of oil grain yield in rapeseed based on field experiments reported in the literature (1-8 in reference).

| Relative planting density of rapeseed to Chinese milk vetch | Oil grain yield (kg ha^-1^) | | Effect size of oil grain yield in rapeseed |
| --- | --- | --- | --- |
|  | Monocropping | Intercropping |  |
| 0.50 | 1666 | 1775 | 0.063 |
| 0.50 | 1112 | 1408 | 0.236 |
| 0.83 | 1995 | 1995 | 0.000 |
| 1.00 | 1995 | 1995 | 0.000 |
| 2.00 | 1995 | 2025 | 0.015 |
| 2.25 | 1276 | 665 | -0.651 |
| 2.25 | 1280 | 1060 | -0.188 |
| 3.00 | 1276 | 1286 | 0.008 |
| 3.00 | 1276 | 910 | -0.337 |
| 3.00 | 1280 | 970 | -0.277 |
| 3.00 | 1280 | 910 | -0.341 |
| 3.20 | 1280 | 980 | -0.267 |
| 3.75 | 1995 | 1950 | -0.022 |
| 3.75 | 1276 | 865 | -0.388 |
| 4.00 | 1280 | 1090 | -0.161 |
| 4.00 | 1276 | 965 | -0.278 |
| 4.50 | 1280 | 830 | -0.433 |
| 4.50 | 1275 | 900 | -0.348 |
| 5.00 | 1280 | 1050 | -0.198 |
| 5.00 | 1276 | 825 | -0.435 |
| 6.00 | 1280 | 770 | -0.508 |
| 6.00 | 1280 | 1040 | -0.207 |
| 7.50 | 1995 | 1980 | -0.008 |
| 7.50 | 1276 | 765 | -0.511 |
| 7.50 | 1276 | 975 | -0.268 |

**References**

1. Cheng Y. 2016. Effects of different planting methods of winter green manure on production efficiency, nutrient utilization and soil fertility in paddy field. Wuhan: Master dissertation Huazhong Agricultural University.
2. Meng ZW, Bai YH, Sang YS, Xu SQ, Zhou KJ, Xu CB, Wu SL. 2005. Study on the Interaction between the Mixed Cropping of Rape and Alfalfa under Non-tillage Seeding and P-fertilizer. *Journal of Anhui Agricultural Sciences* 33: 228-230.
3. Wei YX. 2013. Study on cultivation and utilization of the mixture cropping of Chinese milk vetch with rape and ryegrass. Wuhan: Master dissertation Huazhong Agricultural University.
4. Song L. 2016. Study on the effect of intercropping of rape/Chinese milk vetch and decomposing of their mixture. Wuhan: Master dissertation Huazhong Agricultural University.
5. Song L, Han S, Xi YY, Lu JW, Wu LS, Cao WD, Geng MJ. 2014. Effects of intercropping on growth and yield of rape and Chinese milk vetch. *Chinese Journal of Oil Crop Science* 36: 231-237.
6. Wu SL, Zhou KJ. 2008. Effect of plant density of mixcropping system between rape and milk vetch. *Journal of Crops* 2: 57-59.
7. Xi YY. 2014. Effects of green manure species and planting methods on rice yield, nutrient uptake and soil fertility. Wuhan: Master dissertation Huazhong Agricultural University.
8. Xiong Y, Zhai GD, Ma HQ. 2014. The effects of mixed milk vetch and rape with different ratios in rice field. *Hubei Agricultural Sciences* 53: 5677-5679.
9. Hedges LV, Gurevitch J, Curtis PS. 1999. The meta-analysis of response ratios in experimental ecology. *Ecology* 80: 1150-1156
